# Friend leukemia virus integration 1 promotes tumorigenesis of small cell lung cancer cells by activating the miR-17-92 pathway

**DOI:** 10.18632/oncotarget.16715

**Published:** 2017-03-30

**Authors:** Lingyu Li, Wei Song, Xu Yan, Ailing Li, Xiaoying Zhang, Wei Li, Xue Wen, Lei Zhou, Dehai Yu, Ji-Fan Hu, Jiuwei Cui

**Affiliations:** ^1^ Cancer Center, The First Bethune Hospital of Jilin University, Changchun, China; ^2^ Stanford University Medical School, Palo Alto Veterans Institute for Research, Palo Alto, CA, USA; ^3^ Stanford University Medical School, Palo Alto Veterans Institute for Research, Palo Alto, CA, USA

**Keywords:** FLI1, lung cancer, miR-17-92, tumor, apoptosis

## Abstract

Small cell lung cancer (SCLC) is regarded as the most devastative type of human lung malignancies. The rapid and disseminated growth pattern remains the primary cause of poor clinical prognosis in patients with SCLC. However, the molecular factors that drive rapid progression of SCLC remain unclear. Friend leukemia virus integration 1 (*FLI1*), an Ets transcription factor family member, has been previously reported to act as a major driver of hematological malignancies. In this study, we explored the potential role of *FLI1* in SCLC. Using immunohistochemical staining, we found that *FLI1* was significantly upregulated in SCLC tissues, compared to that in non-small cell lung cancer (NSCLC) and normal lung tissues (*p* < 0.01). The expression score of *FLI1* oncoprotein was associated with the extensive stage of SCLC and the overexpressed Ki67. Knockdown of *FLI1* with small interfering RNA (siRNA) or short hairpin RNA (shRNA) promoted apoptosis and induced repression of cell proliferation, tumor colony formation and *in vivo* tumorigenicity in highly aggressive SCLC cell lines. Importantly, we discovered that *FLI1* promoted tumorigenesis by activating the miR-17-92 cluster family. This study uncovers *FLI1* as an important driving factor that promotes tumor growth in SCLC through the miR-17-92 pathway. *FLI1* may serve as an attractive target for therapeutic intervention of SCLC.

## INTRODUCTION

Small cell lung cancer (SCLC) is the most devastative type of human lung malignancies [1], accounting for approximately 15% of all lung cancer cases recorded [2]. Despite a high initial response rate to first-line chemotherapy, recurrence arises rapidly in the vast majority of cases, usually killing the patient within only a few months. Molecular targeting therapies, although being successful in the treatment of various tumors, often fail in SCLC, primarily because mechanisms of the disease remain unclear [3–5]. It has been shown that bi-allelic inactivation of *TP53* and *RB1* occurs in nearly all the SCLC tumors, and loss of these two tumor suppressors are obligatory in the tumorigenesis and development of SCLC [6]. However, these genetic lesions alone are not sufficient to initiate tumor formation as demonstrated in the model of mouse erythroleukemia [7]. Thus, there must be some molecular regulators that play a vital role in the tumorigenesis of SCLC, in addition to the loss of *TP53* and *RB1*. Clearly, identification of these initial factors is urgently needed to bring breakthroughs to the prognosis assessment and treatment of SCLC.

ETS transcription factors play important roles in cellular proliferation, differentiation, transformation, and apoptosis, and they are correlated with poor survival in some types of cancers [8–10]. Friend leukemia virus integration 1 (*FLI1*), an ETS transcription factor family member, was first identified as a target of proviral integration in F-MuLV-induced mouse erythroleukemia [11]. *FLI1* is preferentially expressed in hematopoietic cells and tissues, endothelial cells and fibroblasts [12, 13], and it has been previously reported to act as a major driver of hematological malignancies [14–17]. Recent studies, however, have documented that *FLI1* is also aberrantly expressed in several solid tumors, including Ewing sarcoma [18], metastatic melanomas [19] and nasopharyngeal carcinoma [18].

Generally, *FLI1* regulates the biological processes of cells through multiple target genes, including *Bcl-2* [14], to inhibit apoptosis in tumorigenesis [20]. An in-depth study also demonstrates that *FLI1* is able to induce apoptosis and growth arrest through the activation of *Caspase3* in some primary Ewing sarcoma cells [21]. Thus, the role of *FLI1* in the regulation of apoptosis may be cell-type dependent. However, little attention has been paid towards the potential role of *FLI1* in SCLC.

In this study, we evaluated the oncogenic role of *FLI1* in the development of SCLC. We were particularly focused on the potential network that is associated with the oncogenic function of *FLI1*. Aberrant expression of *FLI1* in SCLC tissues may serve as a potential target for therapeutic intervention of the disease.

## RESULTS

### Aberrant expression of FLI1 in SCLC tissues

**Figure 1:**
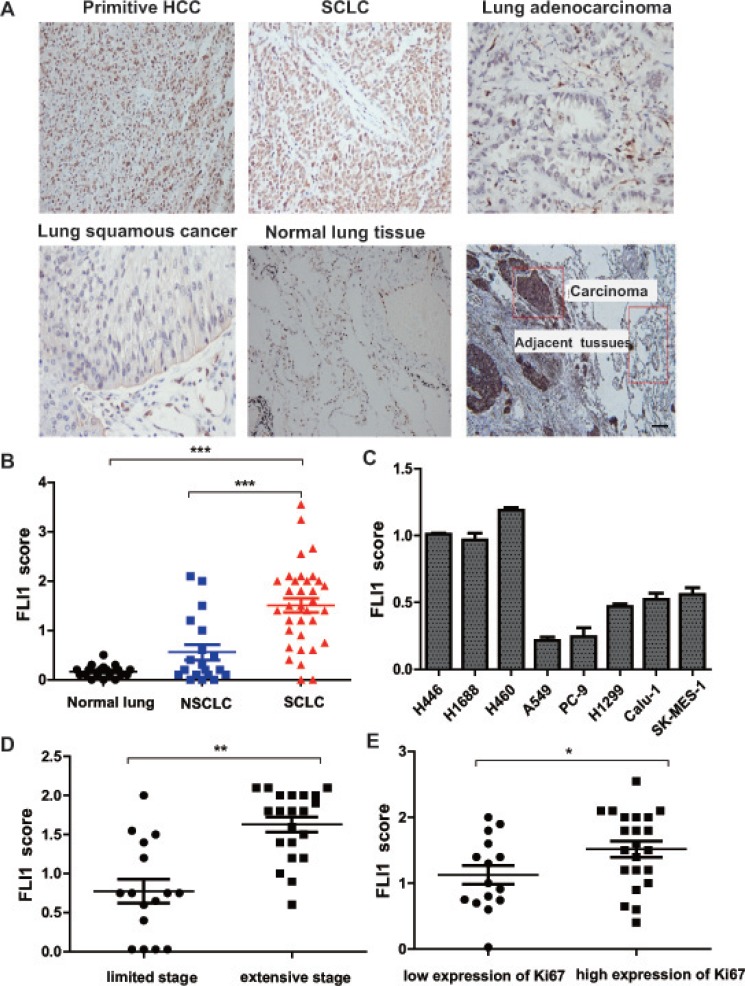
Aberrant expression of *FLI1* in SCLC tissues (**A**) Representative images of *FLI1* immunohistochemical staining in primitive HCC, SCLC, lung adenocarcinoma, lung squamous cancer, normal lung tissue, and one SCLC sample with its carcinoma and adjacent tissue, respectively. (**B**) *FLI1* expression score in normal lung, NSCLC, and SCLC. ***p < 0.001 in SCLC tissues as compared with NSCLC and normal lung tissues. (**C**) Expression of *FLI1* in lung cancer cell lines. SCLC cell lines: NCI-H446 and NCI-H1688; large cell lung carcinoma cell line: NCI-H460; NSCLC cell lines: A549, PC-9, H1299, calu-1 and SK-MES-1 (*p < 0.05). (**D**) *FLI1* scores of SCLC patients in different clinical stages (**p < 0.01). (**E**) *FLI1* expression is positively associated with high expression of Ki67 (*p < 0.05).

We used an immunohistochemical staining approach to identify the correlation between histological type of lung cancer and *FLI1* expression. We compared the expression of FLI1 in 67 SCLC, 20 non-small cell lung cancer (NSCLC), and 20 normal lung specimens (Table 1). FLI1 is a nuclear oncoprotein in hematological malignancies and some solid tumors. Using primitive hepatic cellular carcinoma (HCC) as a positive control, we validated that FLI1 oncoprotein was distributed in the nuclei (Figure 1A, top left panel). Similarly, we found that FLI1 was generally located in the nuclei of SCLC cells with variable intensities (top middle panel). Yet, it was negative or expressed at negligible level in NSCLC (top right and bottom left panels) and normal lung tissues (bottom middle panel). There was a significant difference in expression score between SCLC, NSCLC, and normal tissues (Figure 1B, *p* < 0.0001). Using real time-PCR, we detected the expression of *FLI1* mRNA in lung cancer cell lines. Among the cell lines tested, SCLC cell lines (NCI-H446 and NCI-H1688) and large cell lung carcinoma cell line (NCI-H460) expressed considerably high levels of *FLI1* (Figure 1C).

**Table 1:** Clinical characteristics of patients with SCLC

Variable	Number (%)	Percentage (%)
Age median (range)	57 (47–70)	
≤ 50	24	35.8
>50	43	64.2
Gender		
Male	30	44.8
Female	37	55.2
Stage		
Limited	50	74.6
Extensive	17	25.4
P53 expression		
Positive	59	88
negative	8	12
Ki-67 expression		
Positive	56	83.6
Negative	11	16.4

When categorized based on clinic stages, SCLC patients with extensive stage had significantly higher FLI1 expression score than those with limited stage (*p* < 0.01, Figure 1D). Interestingly, high FLI1 expression score tended to be associated with the over-expression of Ki67 (*p* < 0.05, Figure 1E). We also examined whether *FLI1* could be used as a marker to predict clinical outcomes of SCLC. The results showed that SCLC patients with low expression of FLI1 (scores < 1.25) had a significantly better overall survival (OS) than those with high expression of FLI1 (scores ≥ 1.25). The low FLI1 expression group had significantly longer survival (21.5 months) than did the high FLI1 expression group (12 months) (*p* = 0.036, Supplementary Figure 1).

### FLI1 knockdown inhibits proliferation of SCLC cells

**Figure 2:**
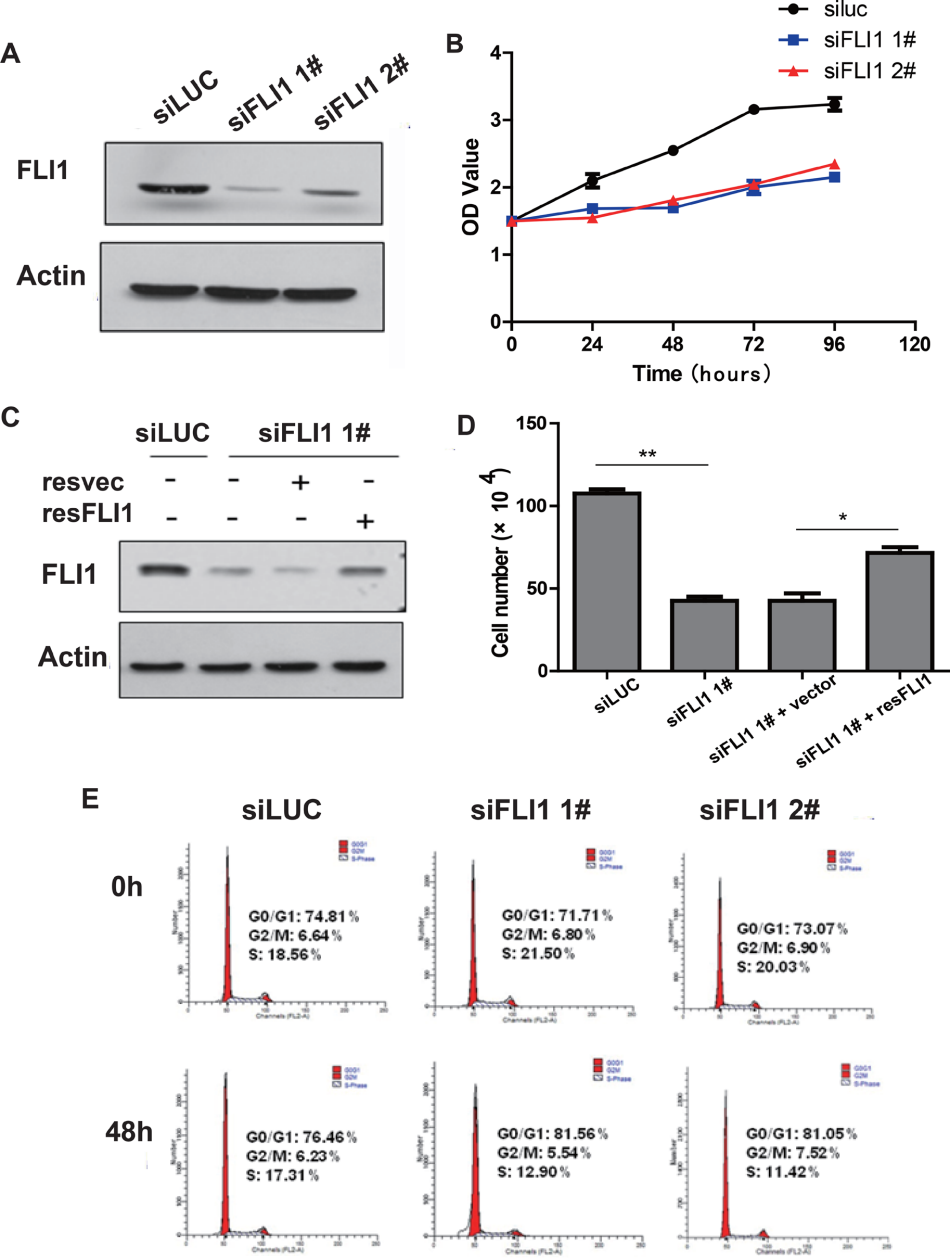
*FLI1* knockdown inhibits proliferation of SCLC cells (**A**) Knockdown of *FLI1* by two siRNAs (si*FLI1* 1#, si*FLI1* 2#) in NCI-H446 cells as measured by Western blot. siLUC: control siRNA targeting the photinuspyralis luciferase genes. si*FLI1*, 2: siRNA targeting *FLI1*. (**B**) Inhibition of cell proliferation by *FLI1* siRNAs in NCI-H446 cells. Cell proliferation is measured by the MTT assay. (**C**) *FLI1* expression in the rescued SCLC cells. The expression of FLI1 oncoprotein is measured by Western blot. (**D**) Re-expression of *FLI1* rescues the proliferation inhibition induced by *FLI1* knockdown. (**E**) Cell cycle in NCI-H446 cells treated with the control siRNA (siLUC) and *FLI1* siRNAs (si*FLI1* 1# and si*FLI1* 2#).

To examine the impact of *FLI1* on biological behavior of SCLC, we used two small interference RNAs (siFLI 1# and siFLI 2#) to knockdown *FLI1* in NCI-H446 cells that express abundant *FLI1*. Efficient knockdown of FLI1 was verified by Western blot (Figure 2A).

The MTT assay showed that the proliferation was significantly reduced inNCI-H446 cells treated with si*FLI1* 1# and si*FLI1* 2# compared to that treated with the siLUC control (Figure 2B, *p* < 0.05). Knockdown of *FLI1* also inhibited cell proliferation in a second SCLC cell line NCI-H1688 (Supplementary Figure 2).

To demonstrate that the change in proliferation was derived from the knockdown of *FLI1*, rather than the “off target” effect of siRNA, si*FLI1*1# (target for the 3′UTR of *FLI1* mRNA) was used for the rescue experiment. The NCI-H446 cells infected with si*FLI1*1# were transiently transfected with a plasmid vector expressing *FLI1*. The re-expression of *FLI1*significantly increased the number of cell proliferation in the NCI-H446 si*FLI1*1# cells as compared with NCI-H446 si*FLI1* 1# cells transfected with the vector control (Figure 2C–2D, *p* < 0.05).

We also analyzed cell cycle in NCI-H446 cells using flow cytometry. After *FLI1* knockdown with siRNA for 48 h, there was a decrease of cells in S-phase (21.5% vs. 12.9% in si*FLI1* 1# and 20.03% vs. 11.42% in si*FLI1* 2#). In parallel, there was a significant increase in blockage of cells in G0/G1-phase (71.71% vs. 81.56% in si*FLI1* 1# and 73.07% vs. 81.05% in si*FLI1* 2#) (Figure 2E). Similar data were also obtained in NCI-H1688 SCLC cells (Supplementary Figure 3). These data suggest that knockdown of *FLI1* may inhibit the proliferation of NCI-H446 cells, at least partially by arresting cell cycle at G0/G1 phase.

### FLI1 knockdown promotes cell apoptosis

**Figure 3:**
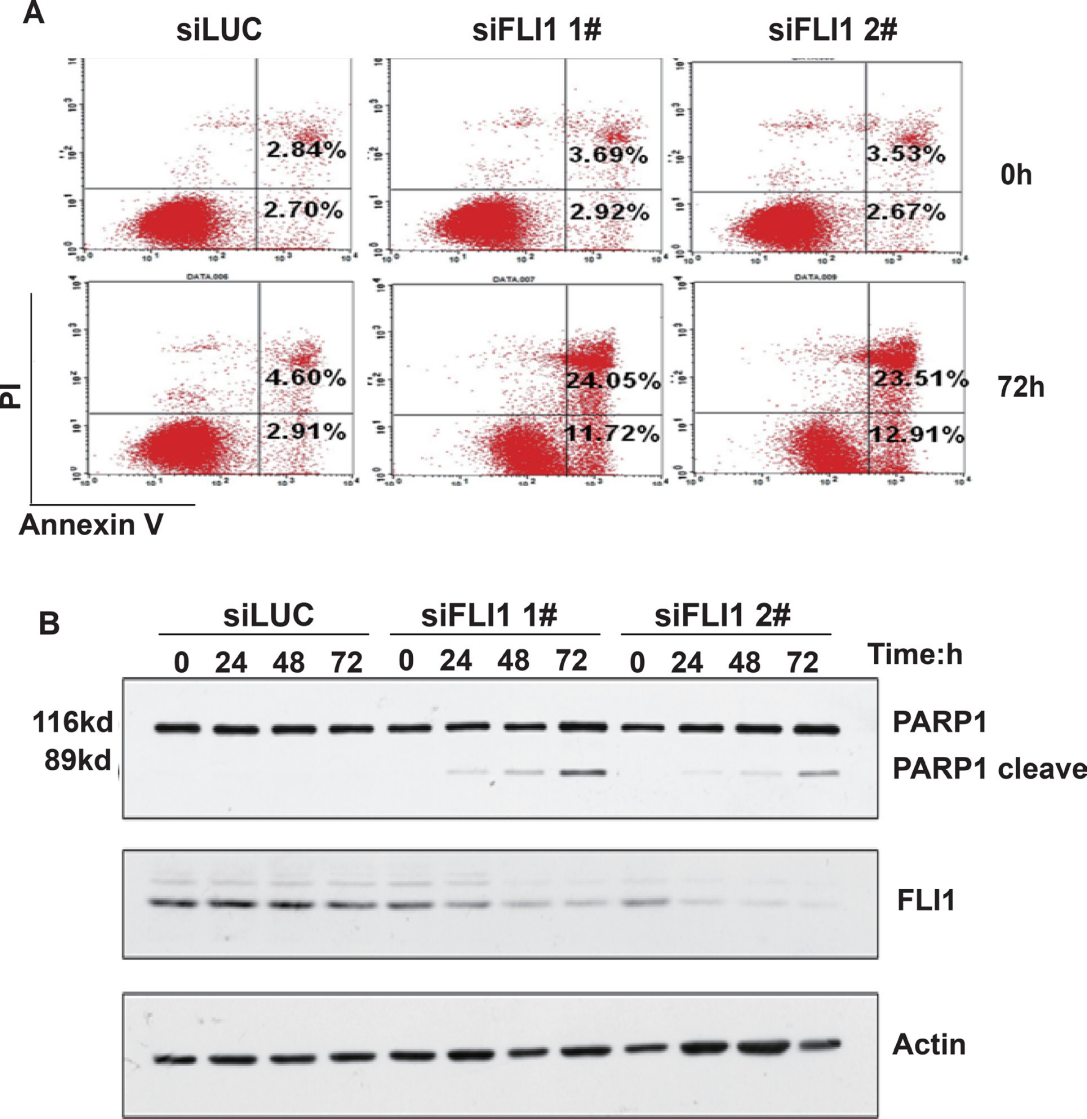
*FLI1* knockdown promotes cell apoptosis (**A**) Apoptosis in NCI-H446 cells. After knockdown of *FLI1*, cells were subject to flow cytometry analysis for cell apoptosis. (**B**) Verification of cell apoptosis by the 89 kd C-terminal fragment of poly ADP-ribose polymerase (PARP). The release of the 89 kd C-terminal fragment is measured by Western blot.

To examine the involvement of apoptosis in the si*FLI1*-treated cells, Annexin V-FITC labeling was performed after 72 h. The apoptotic fraction for the control group with siLUC at 72 h was 2.91%, while the knockdown of *FLI1* led to a dramatic increase in the percentage of apoptotic cells (11.72% in si*FLI1* 1# and 12.91% in si*FLI1* 2#) (Figure 3A). Knockdown of *FLI1* also induced apoptosis in NCI-H1688 cells (Supplementary Figure 4).

We also verified cell apoptosis by examining the 89 kd C-terminal fragment of poly ADP-ribose polymerase (PARP). Western blot showed that the 89 kd cleaved product of PARP was increased in NCI-H446 cells treated with si*FLI1*, compared to those treated with siLUC (Figure 3B).

### FLI1 knockdown inhibits colony formation and tumorigenicity of SCLC cells

**Figure 4:**
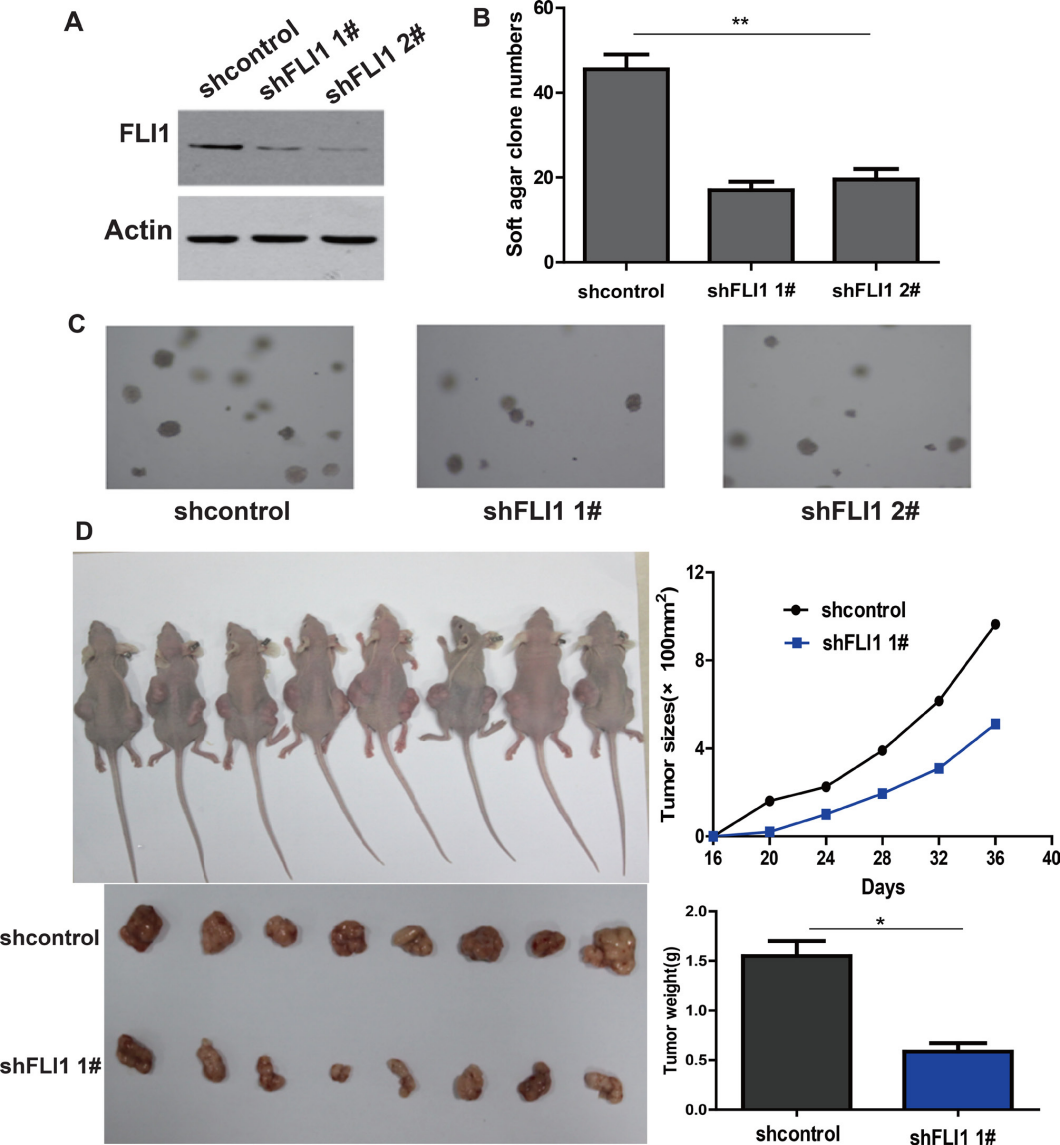
*FLI1* knockdown inhibits tumorigenicity of SCLC cells (**A**) Knockdown of ***FLI1*** by two shRNAs (sh***FLI1*** 1#, sh***FLI1*** 2#) in NCI-H446. *FLI1* expression is measured by Western blot. sh*FLI1* 1#, 2#: shRNA targeting *FLI1*; shControl: control shRNA. (**B**–**C**) The ability of colony formation in NCI-H446 cells following the knockdown of *FLI1*. (**D**) Tumor size and weight in NCI-H446 cells treated with the control shRNA and *FLI1* shRNAs (sh*FLI1* 1#) in nude mouse tumorigenicity assay. *p < 0.05 as compared with the shControl group.

To determine whether *FLI1* is functionally important in tumorigenesis, we knocked down *FLI1* with two different shRNA lentiviruses (sh*FLI1*1# and sh*FLI1* 2#). The knockdown of *FLI1* was confirmed by Western blot in the treated cells (Figure 4A). Compared to the shRNA control, knockdown of *FLI1* significantly inhibited tumor colony formation (*p* < 0.05, Figure 4B–4C).

In a nude mouse tumorigenicity model, we found that the size and weight of tumors were significantly lower in the H446-sh*FLI1* group than that in the H446-shRNA control group (*p* < 0.05, Figure 4D).

### FLI1 promotes cell proliferation by activating the miR-17-92 cluster family

**Figure 5:**
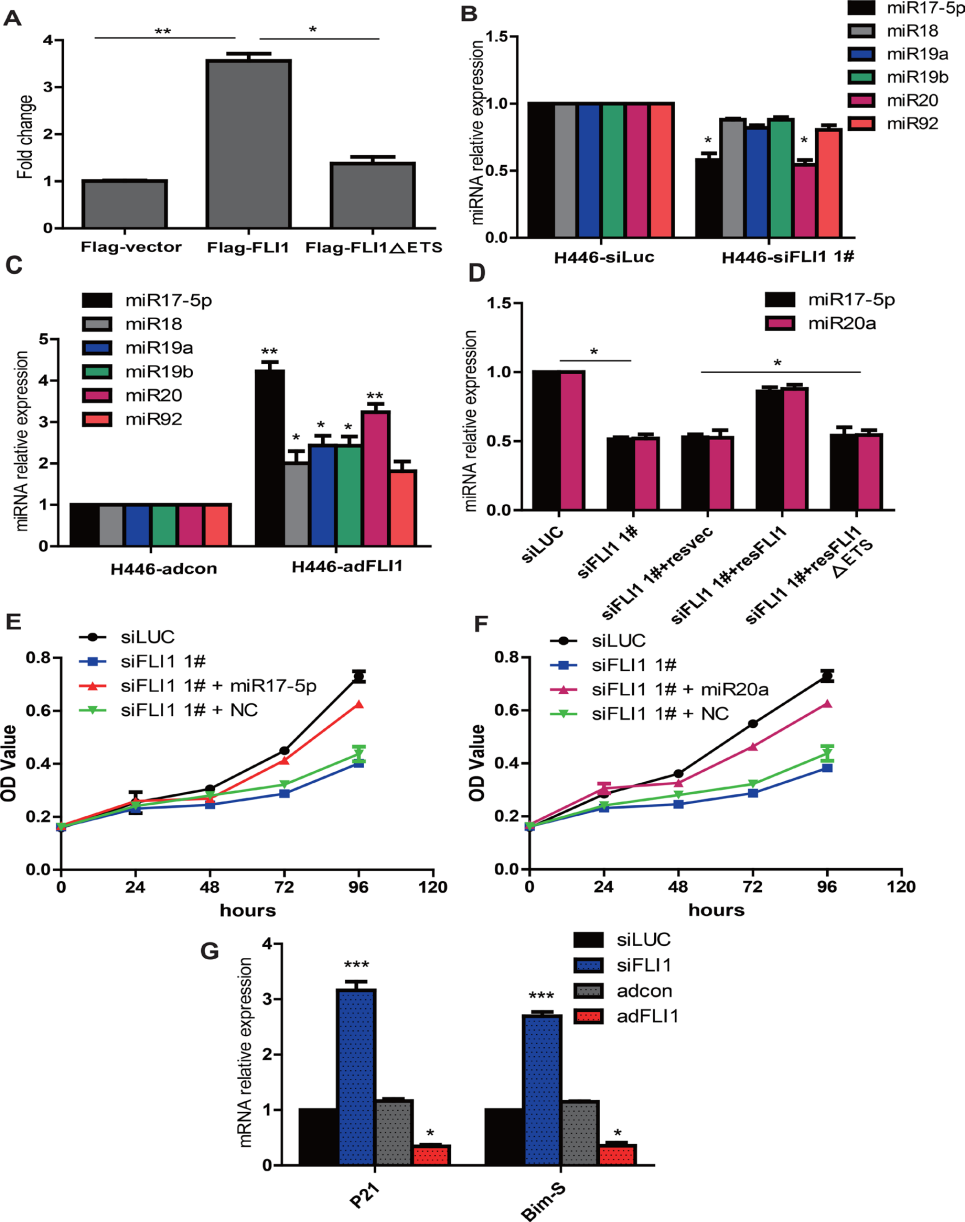
*FLI1* promotes cell proliferation byactivating the miR-17-92 cluster family (**A**) ***FLI1***-luciferase reporter assay. The miR-17-92 promoter-luciferase reporter vector was co-transfected into SCLC cells with ***FLI1***, ***FLI1*** mutant, and vector control plasmid DNAs, respectively. Only Flag-*FLI1* is able to activate transcription of miR-17-92 cluster promoter. (**B**) Expression of 17-92 miRNAs in si*FLI1*-treated NCI-H446 cells. Among the 17-92 miRNA family, miR-17-5p and miR-20a were significantly downregulated by si*FLI1*. *p < 0.05 as compared with the siLuc control group. (**C**) *FLI1* upregulates the 17-92 miRNAs. *p < 0.05 as compared with the adcon control group. (**D**) *FLI1* rescues the down-regulation of miR-17-5p and miR-20a induced by *FLI1* knockdown.*p < 0.05 as compared with the si*FLI1* group. (**E**) miR-17-5p mimics rescue the proliferation inhibition induced by *FLI1* knockdown. (**F**) miR-20a mimics rescue the proliferation inhibition induced by *FLI1* knockdown. (**G**) *FLI1* controls the miR-17-92 downstream genes *P21* and *Bim-S*. *p < 0.05 and ***p < 0.01 as compared with the siLuc control group. The miR-17-5p/miR-20a downstream target genes (*P21* and *Bim-S*) are upregulated by si*FLI1* knockdown, but are downregulated by ad*FLI1* expression.

The miR-17-92 family is one of the best-known miRNA clusters involved in hematopoietic and solid cancers, including SCLC. We thus examined whether *FLI1* functions by targeting the miR-17-92 cluster. For this, we first constructed the miR-17-92 promoter/luciferase reporter. The luciferase vectors were co-transfected into HEK-293T cells with Flag-*FLI1*, Flag-*FLI1∆* ETS (mutation in the ETS domain), and Flag-vector (control). Ectopic expression of *FLI1* significantly increased the activity of luciferase reporter by at least 3 times, compared to the vector control (Figure 5A). The Flag-*FLI1*∆ETS construct that carry a mutation in the ETS domain lacked the transactivator activity, suggesting that *FLI1* may activate the miR-17-92 cluster through its ETS domain.

To further delineate the underlying mechanism of *FLI1*, we examined the expression of mature 17-92 miRNAs in treated SCLC cells, including miR-17-5p, miR-18a, miR-19a, miR-19b, miR-20a and miR-92a. In the 17-92 miRNA family, miR-17-5p and miR-20a were closely associated with the expression of *FLI1*, being downregulated in the si*FLI1*-treated cells (*p* < 0.05, Figure 5B) and upregulated in the *FLI1*-expressing cells (*p* < 0.05, Figure 5C).

To confirm the regulation of *FLI1* on miR-17-5p and miR-20a, we used the re-expression of Flag-*FLI1*, Flag-*FLI1*∆ETSor Flag-vector to rescue the down-regulation of miR-17-5p and miR-20a expression induced by *FLI1* knockdown. We showed that Flag-*FLI1*, rather than the Flag-*FLI1*∆ETS mutant or the Flag-vector control, was able to rescue the downregulated miR-17-5p and miR-20a (*p* < 0.05, Figure 5D).

Both of miR-17-5p and miR-20a had been found to serve as growth-promoting miRNAs in SCLC [22]. We investigated whether miR-17-5p and miR-20a mimics could rescue the inhibition of cell proliferation induced by *FLI1* knockdown. Importantly, we found that the inhibition of cell proliferation was rescued efficiently by both miRNA mimics (Figure 5E, miR-17-5p; Figure 5F, miR20-a).

Finally, we examined whether the downstream target genes of miR-17-5p and miR-20a were also affected by *FLI1*. When the suppressor effect of miR-17-92 was removed in the si*FLI1*-treated cells, the expression of *Bim-S* and CDKN1A (*P21*) was significantly increased (Figure 5G). In contrast, adenoviral expression of *FLI1* transactivated miR-17-92, leading to the suppression of both downstream target genes. Using Western blot, we also confirmed the altered expression of multiple downstream pathway genes by overexpression or knockdown of *FLI1* (Supplementary Figure 5). Collectively, our data identify *FLI1* as an upstream component of the miR-17-92 pathway in SCLC.

## DISCUSSION

The rapid and disseminated growth pattern remains the primary cause of poor clinical prognosis in patients with SCLC. Thus, identification of genetic determinants involved in the biological processes of SCLC is significant for evaluating prognosis and guiding therapeutic intervention. In this study, we demonstrate that *FLI1* is an underlying oncogenic gene in tumorigenesis and development of SCLC. The oncogenic factor promotes tumorigenesis in SCLC by activating the miR-17-92 pathway.

It is well known that oncogenic activation of *FLI1* leads to tumorigenesis, such as Ewing sarcoma and ovarian cancer. However, little is known about the role of *FLI1* in SCLC. To investigate the role of *FLI1* in SCLC, we first examined the expression of *FLI1* in lung cancer by immunohistochemistry staining. We showed that *FLI1* was overexpressed in SCLC compared to adjacent/normal and NSCLC tissues. Clinicopathologic analysis revealed that the high expression of FLI1 oncoprotein was positively correlated with extensive stage. Overexpression of *FLI1* in patients with SCLC was tended to be associated with higher positive expression rate of Ki-67. Using two SCLC cell lines as a model, we also found that knockdown of *FLI1* with siRNA or shRNA promoted apoptosis and led to inhibition of cell proliferation, tumor colony formation and *in vivo* tumorigenicity. Taken together, our data suggest a critical role of *FLI1* in tumorigenesis of SCLC.

*FLI1*, as an Ets family member of transcription factors, was identified as a proto-oncogene in other tumors through the regulation of different target genes, including upregulation of *Bcl-2* to inhibit apoptosis in tumor cells, transcriptional downregulation of *gata-1* and *RB1* to inhibit erythroid differentiation, and direct upregulation of *MDM2* to destabilize the anti-apoptotic protein TP53 in tumor progression [23–26]. It is known that several known tumor suppressors that are negatively regulated by *FLI1*, such as *TP53* and *RB1*, were inactivated in SCLC cells. Thus, *FLI1* cannot play a functional role through these negative signal pathways [6]. Even if *Bcl-2* was over-expressed in the majority of SCLCs, several *Bcl-2* inhibitors, such as G3139, did not improve any clinical outcome of patients with SCLC when they combined with chemotherapy [27]. Obviously, there might be some unknown *FLI1* targets that are involved in the development and progression of SCLC.

Recent studies indicate that dysregulation of miRNAs is implicated in the pathogenesis of cancer and metastasis [28–30]. The miR-17-92 cluster (miR17-5p, miR-18, miR-19a, miR-19b, miR-20a and miR-92a), located in an intron of *MIR17HG* [miR-17-92 cluster host gene (non-protein coding)] on chromosome 13 (13q31.3), is overexpressed in lung cancers, especially with the most aggressive SCLC [31]. Aberrant expression of miR-17-92 plays an important oncogenic role in aggressive SCLC cells through the regulation of downstream target genes, such as *PTEN*, *P21*, *Bim*, and *E2F* [32]. Schwentner et al. found that EWS-FLI1 activated the miR-17-92 cluster in Ewing sarcoma [33]. However, it remains unclear whether *FLI1* may function through the miR-17-92 cluster in SCLC.

In this study, we reveal that FLI1 directly activates the promoter of miR17-92 cluster through the ETS binding domain in human SCLC cells (Supplementary Figure 2). Overexpression of *FLI1* induces its binding to the miR-17-92 promoter and activates the cluster transcription in SCLC cells. In contrast, knockdown of *FLI1* reduces miR-17-92 levels in the same cell line. Two major components of the microRNA cluster, miR-17–5p and miR-20a [34], have been reported to target a noticeable large subset of key genes that promote proliferation and cell cycle progression in SCLC [22]. Using a rescue experiment, we demonstrate that knockdown of *FLI1*, particularly its ETS binding domain, alters the expression of miR-17-5p and miR-20a. Only Flag-*FLI1*, rather than Flag-*FLI1*∆ETS that carry the mutant ETS binding domain, can rescue these two miRNAs induced by *FLI1* knockdown in SCLC cells. Similarly, miR-17-5p and miR-20a mimics could rescue the inhibition of cell proliferation induced by *FLI1* knock-down in SCLC cells.

The miR-17-92 cluster regulates multiple downstream target genes, including *PTEN*, *P21*, *Bim*, and *E2F* [32, 35]. For example, miR-17-5p and miR-20a mainly inhibit the expression of target genes *Bim-S* and *P21* [35, 36]. We also found that two miR-17-92 downstream targets, *Bim-S* and *P21*, were also affected by FLI1 in our model. Thus, our data suggest a novel *FLI1*/miR-17-92/*Bim-S-P21* pathway in SCLC. In this pathway, the oncogenic *FLI1* regulates miR-17-5p and miR-20a through its binding to a conserved ETS binding site in the gene promoter. In supporting this, we recently show that FLI1 is also involved in transcriptional activation of miR-17-92 in mouse erythroleukemic cells [37].

In conclusion, this study for the first time to uncover that *FLI1* influences tumorigenesis and cellular proliferation of SCLC cells by regulating the miR-17-92 cluster transcription, especially miR-17-5p and miR-20a. After knocking down *FLI1*, both miR-17-5p and miR-20a are significantly downregulated, releasing their downstream suppression targets *P21* and *Bim-S*, leading to reduced cell proliferation, enhanced apoptosis, and finally the suppression of tumors (Supplementary Figure 6). Thus, this study identifies *FLI1* as an attractive target for therapeutic intervention in SCLC.

## MATERIALS AND METHODS

### Parents and samples

Formalin-fixed paraffin-embedded tissues of lung cancer and control samples were obtained from the First Hospital of Jilin University between 2005 and June 2011. Primitive hepatic angiosarcoma was chosen as the positive control. Clinical data related to disease stage and histological grade were available for these patients. The pathological diagnosis was made in accordance with the histological classification of tumors developed by the World Health Organization. Tumor stage was defined according to American Joint Committee on Cancer/International Union Against Cancer classification system. Tumors were histologically graded according to the Elston and Ellis method. The study was approved by the Research Ethics Board of the First Hospital of Jilin University. Informed consent was obtained from each participant. The clinical characteristics of all patients with SCLC were shown in Table 1.

### Immunohistochemical staining

Tissue slides were obtained from the First Hospital of Jilin University. Slides were de-paraffinized with xylene and rehydrated through a gradual decline of alcohol (100–80%), and then incubated in 3% hydrogen peroxide for 15 minutes to block endogenous peroxidase activity. Antigen retrieval was carried out by immersing the slides in 10 mM sodium citrate buffer (pH 6.0) and maintained at a sub-boiling temperature for 15 minutes. The slides were rinsed in phosphate-buffered saline and incubated with 10% normal goat serum to block non-specific staining for 30 minutes at 37°C. Primary anti-FLI1 poly-clonal antibodies (Neomarker) were diluted in 1:100, and incubated with the sections at 4°C overnight. After washing with PBS, the secondary antibodies (biotinylated goat anti-rabbit immunoglobulin) and streptavidin peroxidase complex reagent were applied. Subsequently, the visualization signal was processed according to the Polink-2 HRP DAB Detection kit. Finally, the slides were counterstained with hematoxylin for 15 min and dehydrated in ascending concentrations of alcohol (80–100%). After xylol treatment, slides were covered. Two investigators evaluated each stained section independently without knowing any clinical information. The proportions of positive cells were ranged from 10 to 100%, while the intensity of staining was scored as 0 (negative), 1 (weak), 2 (moderate), and 3 (intense) in the most strongly stained tumor area. The immunoreactivity score for each case was taken as percentage of positive cells multiplied by the intensity of staining [IHC score = Σ (intensity) × % positive cells].

### MiRNA and siRNA transfection

H446 cells were plated in 12-well plates at 2 × 10^5^ per well. Twenty-four hours after plating, 40 pmole of miRNA (Ribobio) or siRNAs against *FLI1* (Invitrogen, CA) were transfected to the cells with Lipofectamine RNAiMax (Invitrogen, CA) following manufacture protocol. Negative miRNA (Ribobio) or siLUC (Invitrogen) was also transfected as negative controls.

### Construction of plasmids

Flag-*FLI1* was obtained by cloning PCR-amplified full-length CDS of the human *FLI1* gene and then inserted into the p3×Flag-CMV plasmid (kindly provided from National Center of Biomedical Analysis, China). The vector p3×Flag-CMVwas double digested with HindIII and Kpn1.Flag-*FLI1*∆ETS(*FLI1*∆ETS) was constructed by deleting codons 281–361(DNA binding domain) from Flag-*FLI1* using oligos F-DBD-*FLI1*-361 and R-DBD-*FLI1*-281.

The luciferase reporter with the inclusion of miR17-92 cluster promoter sequence was constructed by subcloning human miR-17-92 cluster promoter region directly into the upstream of a cytomegalovirus promoter-driven firefly luciferase (FL-miR17-92) cassette in the pCDNA3.0 vector.

Furthermore, two retroviral expression vector of short hairpin RNA (shRNA) against *FLI1* were constructed and transfected into HEK-293 cells to produce virus that can transfer shRNA. The sh*FLI1* 1# sequence was 5′-CGTCATGTTCTGGTTTGAGAT-3′ and sh*FLI1* 2# sequence was 5′ GCACAAACGATCAGTAAGAAT-3′. The reconstructed plasmid was sequence verified viaInvitrogen.

### Cell culture

Lung cancer cell lines (NCI-H446, NCI-H1688, NCI-H460, H1299, A549, PC-9, Calu-1 and SK-MES-1) were obtained from ATCC, and maintained in medium (NCI-H446, NCI-H460, NCI-H1688 and H1299 cell lines were cultured in RPMI-1640, and A549, PC-9, Calu-1, SK-MES-1 and HEK-293 cell lines were cultured in DMEM) supplemented with 10% fetal bovine serum(FBS, Hyclone) and 1% penicillin/streptomycin at 37°C with 5% CO2.

### Cell proliferation assay

For cell proliferation assay, H446cells were seeded in 96-well plates with density 20% per well. The number of cell proliferation was measured by MTT assay from day 1 to day 4. For staining, 100 μl of 0.5 mg/ml MTT was added to each well and cultured in 37– incubator for 4 h. Then, 100 ul DMSO was added to each well after removing media carefully. The absorbance was read at 490 nm. The experiment was repeated three times.

### Cell cycle and apoptosis analysis by flow cytometry

H446 cells were fixed in absolute ethyl alcohol at −20– overnight, washed twice with PBS, and resuspended in PI staining solution containing 0.1 mg/mL RNase A, 50 μg/mL propidium-iodide, and 0.2% Triton. Cell cycle distribution was analyzed using bivariate flow cytometry on a FACSCalibur (BD). FlowJo software (TreeStar) was used for cell cycle position using the cell cycle algorithm.

To detect cell apoptosis, H446 cells were seeded in 12-well plates with density 20% per well, resuspended in 500 ul Annexin V binding buffer, and incubated with 5 μl Annexin V-FITC for 10 min and 5 μl PI for 15 min, following the manufacture's instruction by flow cytometry.

### Soft agar assays

In each experiment, 5 × 10^3^ H446 cells were trypsinized and resuspended in 3ml of complete medium (20% FBS) in 0.3% agarose LMP (Invitrogen). After incubation at 37°C for 2 weeks, the agar assays were scored for viable colonies.

### Nude mouse tumorigenicity assay

BALB/c female nude mice (age, 6–8 weeks) were purchased from Vital River Laboratory Animal Technology Co. Ltd. (Beijing, China) under specific pathogen-free (SPF) conditions. A self-control model was used to examine the role of *FLI1* in tumorigenicity. Each mouse was inoculated subcutaneously (s.c.) with equal amount (5 × 10^6^) of *FLI1*-positive H446 cells on the left side and *FLI1*-knockdown H446 cells on the right side of backbone in nude mice, respectively. The size of the subcutaneous tumor was regularly measured using a vernier caliper to monitor tumor growth. The tumors were excised in 6 weeks after inoculation.

### The miR-17-92 luciferase reporter assay

Cells were seeded in 24-well plates at a density of 5 × 10^4^ cells per well 24 h before transfection. The cells were co-transfected with a mixture of 100ng FL-miR17-92 reporter vectors, 10ng Renilla luciferase (RL) reporter vectors(pRL-TK), and Flag-*FLI1* or Flag-*FLI1*△ETS. After 48 h, the luciferase activity was measured with a dual luciferase reporter assay system (Promega, Madison, WI). In the luciferase report assay, we used one internal control (RL reporter) and two negative controls (Flag-vector control and luciferase reporter lacking the miR17-92 cluster promoter). For comparison, the FL-miR17-92 activity was first normalized with RL activity. The effect of Flag-vector, Flag-*FLI1* or Flag-*FLI1*∆ETS on luciferase reporter with miR17-92 cluster promoter was then normalized with that on luciferase reporter without miR17-92 cluster promoter. Finally, the fold change was calculated by Flag-*FLI1* or Flag-*FLI1*△ETS compared with Flag-vector.

### Western immunoblotting

Cells were lysed in radioimmunoprecipitation assay buffer (5 mM Tris pH 7.4, 1% NP-40, 0.15 M NaCl, 0.1% SDS, plus protease inhibitor cocktail and 1 mM phenylmethylsulfonyl fluoride). Equal amounts of protein were resolved by SDS-PAGE and subjected to Western blot analysis using enhanced chemilluminescence (Pierce). Antibodies to FLI1, PARP1 and β-Actin were obtained from Abcam (Shanghai, China). Antibodies against CyclinE, CDK2, Bcl-2 and P21 were obtained from Santa Cruz Biotechnology (CA, USA).

### RNA preparation and real-time PCR

Total RNA was isolated using Trizol (Life Technologies, Carlsbad, CA).To quantify the amount of mature miRNA, we used TaqMan MicroRNA assays(Life Technologies) and small nuclear U6B (RNU6B) RNA as an internal standard. To quantify the amount of mRNA, cDNA was synthesized with the Prime Script RT Master Mix (Takara, Japan) from 500ng of RNA. The real-time PCR analyses were performed using SYBR Premix Ex Taq II (Takara). The relative expression of each gene was quantified on the basis of CT value measured against an internal standard curve for each specific set of primers and using Biosystems 7300 Real-Time PCR system. These data were normalized to the β-Actin control.

### Statistical analysis

Comparisons between groups were analyzed by *t*-test. Comparison was made of groups with high FLI1 expression (score > median score) and low FLI1 expression (score ≤ median score). We assessed score comparisons between groups by one-way ANOVA test. Overall Survival (OS) were calculated by using the Kaplan-Meier method, and the differences were assessed by using the log-rank test. *p* value of less than 0.05 was considered significant. Statistical calculations were performed using SPSS 13.0.

## SUPPLEMENTARY FIGURES



## References

[1] Ferlay J, Soerjomataram I, Dikshit R, Eser S, Mathers C, Rebelo M, Parkin DM, Forman D, Bray F (2015). Cancer incidence and mortality worldwide: sources, methods and major patterns in GLOBOCAN 2012. Int J Cancer.

[2] Siegel R, Ma J, Zou Z, Jemal A (2014). Cancer statistics, 2014. CA Cancer J Clin.

[3] Jotte R, Conkling P, Reynolds C, Galsky MD, Klein L, Fitzgibbons JF, McNally R, Renschler MF, Oliver JW (2011). Randomized phase II trial of single-agent amrubicin or topotecan as second-line treatment in patients with small-cell lung cancer sensitive to first-line platinum-based chemotherapy. J Clin Oncol.

[4] Schmittel A, Sebastian M, Fischer von Weikersthal L, Martus P, Gauler TC, Kaufmann C, Hortig P, Fischer JR, Link H, Binder D, Fischer B, Caca K, Eberhardt WE (2011). A German multicenter, randomized phase III trial comparing irinotecan-carboplatin with etoposide-carboplatin as first-line therapy for extensive-disease small-cell lung cancer. Ann Oncol.

[5] Spigel DR, Townley PM, Waterhouse DM, Fang L, Adiguzel I, Huang JE, Karlin DA, Faoro L, Scappaticci FA, Socinski MA (2011). Randomized phase II study of bevacizumab in combination with chemotherapy in previously untreated extensive-stage small-cell lung cancer: results from the SALUTE trial. J Clin Oncol.

[6] George J, Lim JS, Jang SJ, Cun Y, Ozretic L, Kong G, Leenders F, Lu X, Fernandez-Cuesta L, Bosco G, Muller C, Dahmen I, Jahchan NS (2015). Comprehensive genomic profiles of small cell lung cancer. Nature.

[7] Wong KS, Li YJ, Howard J, Ben-David Y (1999). Loss of p53 in F-MuLV induced-erythroleukemias accelerates the acquisition of mutational events that confers immortality and growth factor independence. Oncogene.

[8] Maroulakou IG, Bowe DB (2000). Expression and function of Ets transcription factors in mammalian development: a regulatory network. Oncogene.

[9] Davidson B, Reich R, Goldberg I, Gotlieb WH, Kopolovic J, Berner A, Ben-Baruch G, Bryne M, Nesland JM (2001). Ets-1 messenger RNA expression is a novel marker of poor survival in ovarian carcinoma. Clin Cancer Res.

[10] Oikawa T (2004). ETS transcription factors: possible targets for cancer therapy. Cancer Sci.

[11] Ben-David Y, Giddens EB, Bernstein A (1990). Identification and mapping of a common proviral integration site Fli-1 in erythroleukemia cells induced by Friend murine leukemia virus. Proc Natl Acad Sci USA.

[12] Hart A, Melet F, Grossfeld P, Chien K, Jones C, Tunnacliffe A, Favier R, Bernstein A (2000). Fli-1 is required for murine vascular and megakaryocytic development and is hemizygously deleted in patients with thrombocytopenia. Immunity.

[13] Spyropoulos DD, Pharr PN, Lavenburg KR, Jackers P, Papas TS, Ogawa M, Watson DK (2000). Hemorrhage, impaired hematopoiesis, and lethality in mouse embryos carrying a targeted disruption of the Fli1 transcription factor. Mol Cell Biol.

[14] Cui JW, Vecchiarelli-Federico LM, Li YJ, Wang GJ, Ben-David Y (2009). Continuous Fli-1 expression plays an essential role in the proliferation and survival of F-MuLV-induced erythroleukemia and human erythroleukemia. Leukemia.

[15] Tyybakinoja A, Saarinen-Pihkala U, Elonen E, Knuutila S (2006). Amplified, lost, and fused genes in 11q23-25 amplicon in acute myeloid leukemia, an array-CGH study. Genes Chromosomes Cancer.

[16] Kwiatkowski BA, Zielinska-Kwiatkowska AG, Bauer TR, Hickstein DD (2000). The ETS family member Tel antagonizes the Fli-1 phenotype in hematopoietic cells. Blood Cells Mol Dis.

[17] Kwiatkowski BA, Bastian LS, Bauer TR, Tsai S, Zielinska-Kwiatkowska AG, Hickstein DD (1998). The ets family member Tel binds to the Fli-1 oncoprotein and inhibits its transcriptional activity. J Biol Chem.

[18] Johnson AD, Pambuccian SE, Andrade RS, Dolan MM, Aslan DL (2010). Ewing sarcoma and primitive neuroectodermal tumor of the esophagus: report of a case and review of literature. Int J Surg Pathol.

[19] Torlakovic EE, Slipicevic A, Florenes VA, Chibbar R, DeCoteau JF, Bilalovic N (2008). Fli-1 expression in malignant melanoma. Histol Histopathol.

[20] Owen LA, Lessnick SL (2006). Identification of target genes in their native cellular context: an analysis of EWS/FLI in Ewing's sarcoma. Cell Cycle.

[21] Sohn EJ, Li H, Reidy K, Beers LF, Christensen BL, Lee SB (2010). EWS/FLI1 oncogene activates caspase 3 transcription and triggers apoptosis in vivo. Cancer Res.

[22] Matsubara H, Takeuchi T, Nishikawa E, Yanagisawa K, Hayashita Y, Ebi H, Yamada H, Suzuki M, Nagino M, Nimura Y, Osada H, Takahashi T (2007). Apoptosis induction by antisense oligonucleotides against miR-17-5p and miR-20a in lung cancers overexpressing miR-17-92. Oncogene.

[23] Yi H, Fujimura Y, Ouchida M, Prasad DD, Rao VN, Reddy ES (1997). Inhibition of apoptosis by normal and aberrant Fli-1 and erg proteins involved in human solid tumors and leukemias. Oncogene.

[24] Pereira R, Quang CT, Lesault I, Dolznig H, Beug H, Ghysdael J (1999). FLI-1 inhibits differentiation and induces proliferation of primary erythroblasts. Oncogene.

[25] Tamir A, Howard J, Higgins RR, Li YJ, Berger L, Zacksenhaus E, Reis M, Ben-David Y (1999). Fli-1, an Ets-related transcription factor, regulates erythropoietin-induced erythroid proliferation and differentiation: evidence for direct transcriptional repression of the Rb gene during differentiation. Mol Cell Biol.

[26] Athanasiou M, Mavrothalassitis G, Sun-Hoffman L, Blair DG (2000). FLI-1 is a suppressor of erythroid differentiation in human hematopoietic cells. Leukemia.

[27] Lu HY, Wang XJ, Mao WM (2013). Targeted therapies in small cell lung cancer. Oncol Lett.

[28] Bartel DP (2004). MicroRNAs: genomics, biogenesis, mechanism, and function. Cell.

[29] Valastyan S, Weinberg RA (2009). MicroRNAs: Crucial multi-tasking components in the complex circuitry of tumor metastasis. Cell Cycle.

[30] Ventura A, Jacks T (2009). MicroRNAs and cancer: short RNAs go a long way. Cell.

[31] Hayashita Y, Osada H, Tatematsu Y, Yamada H, Yanagisawa K, Tomida S, Yatabe Y, Kawahara K, Sekido Y, Takahashi T (2005). A polycistronic microRNA cluster, miR-17-92, is overexpressed in human lung cancers and enhances cell proliferation. Cancer Res.

[32] Osada H, Takahashi T (2011). let-7 and miR-17-92: small-sized major players in lung cancer development. Cancer Sci.

[33] Schwentner R, Herrero-Martin D, Kauer MO, Mutz CN, Katschnig AM, Sienski G, Alonso J, Aryee DN, Kovar H (2017). The role of miR-17-92 in the miRegulatory landscape of Ewing sarcoma. Oncotarget.

[34] Ebi H, Sato T, Sugito N, Hosono Y, Yatabe Y, Matsuyama Y, Yamaguchi T, Osada H, Suzuki M, Takahashi T (2009). Counterbalance between RB inactivation and miR-17-92 overexpression in reactive oxygen species and DNA damage induction in lung cancers. Oncogene.

[35] Fuziwara CS, Kimura ET (2015). Insights into Regulation of the miR-17-92 Cluster of miRNAs in Cancer. Front Med (Lausanne).

[36] Weng H, Huang H, Dong B, Zhao P, Zhou H, Qu L (2014). Inhibition of miR-17 and miR-20a by oridonin triggers apoptosis and reverses chemoresistance by derepressing BIM-S. Cancer Res.

[37] Cui JW, Li YJ, Sarkar A, Brown J, Tan YH, Premyslova M, Michaud C, Iscove N, Wang GJ, Ben-David Y (2007). Retroviral insertional activation of the Fli-3 locus in erythroleukemias encoding a cluster of microRNAs that convert Epo-induced differentiation to proliferation. Blood.

