# Supplementary material for: Friend leukemia virus integration 1 promotes tumorigenesis of small cell lung cancer cells by activating the miR-17-92 pathway
Source: Oncotarget. 2017 Mar 30;8(26):41975–87. doi: 10.18632/oncotarget.16715 (PMC5522042; doi:10.18632/oncotarget.16715)
Supplement: Supplementary file 1 [file oncotarget-08-41975-s001.pdf]

Friend leukemia virus integration 1 promotes tumorigenesis of small cell lung cancer cells by activating the miR-17-92 pathway

Supplementary Materials

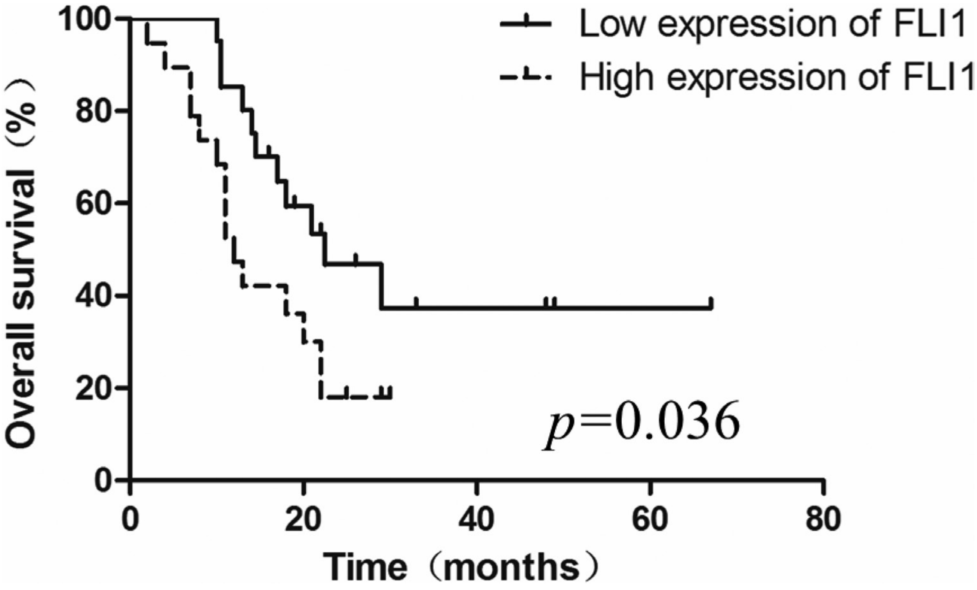

Supplementary Figure 1: *FLI1* is a potential biomarker for the survival of SCLC. The overall survival of SCLC was analyzed by Kaplan-Meier estimates. SCLC patients with low expression of *FLI1* had better OS than those with high expression of *FLI1* ($p = 0.036$).

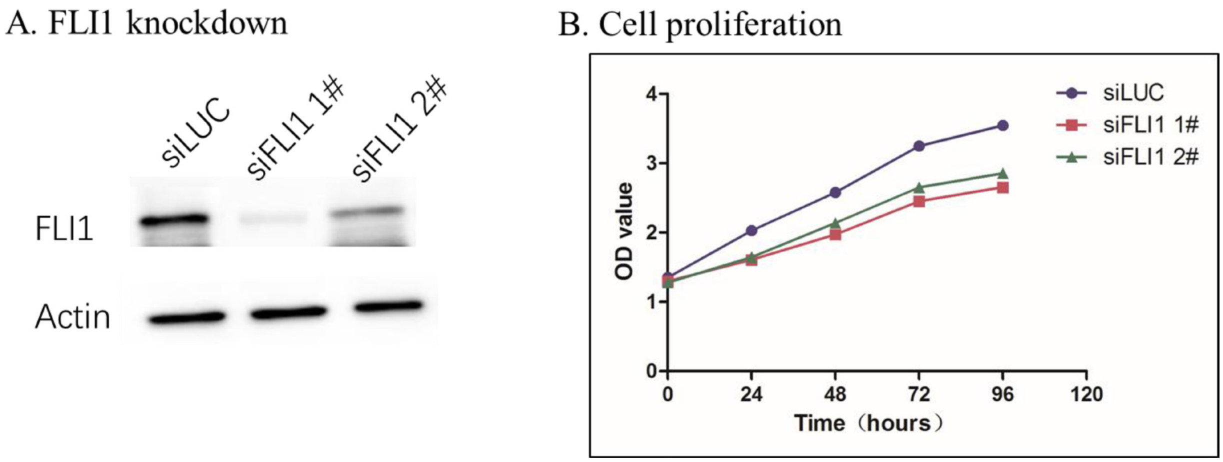

Supplementary Figure 2: *FLI1* knockdown inhibits cell proliferation in NCI-H1688 SCLC cells. (**A**) Knockdown of *FLI1* by two siRNAs (si*FLI1* 1#, si*FLI1* 2#) in NCI-H1688 cells. Expression of *FLI1* was measured by Western blot. siLUC: control siRNA that targets the photinuspyralis luciferase genes; si*FLI1*: siRNAs that target *FLI1*. (**B**) Inhibition of cell proliferation by *FLI1* siRNAs in NCI-H1688 cells. Cell proliferation is measured by the MTT assay.

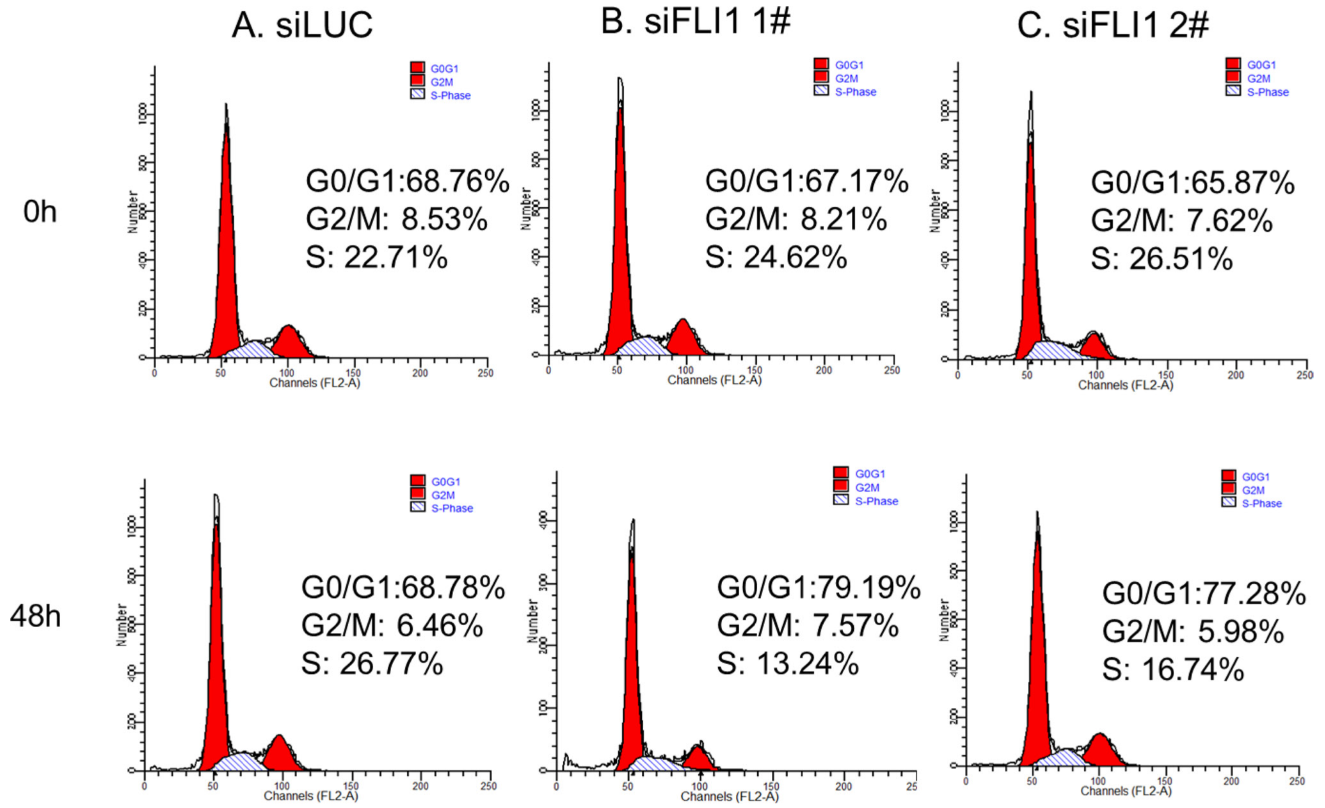

Supplementary Figure 3: ***FLI1*** **knockdown induces G0/G1 cell cycle arrest in NCI-H1688 cells Cell cycle was examined by flow cytometry (FACS) in NCI-H1688 cells treated with the control siRNA (siLUC) and** ***FLI1*** **siRNAs (si*****FLI1*** **1# and si*****FLI1*** **2#).**

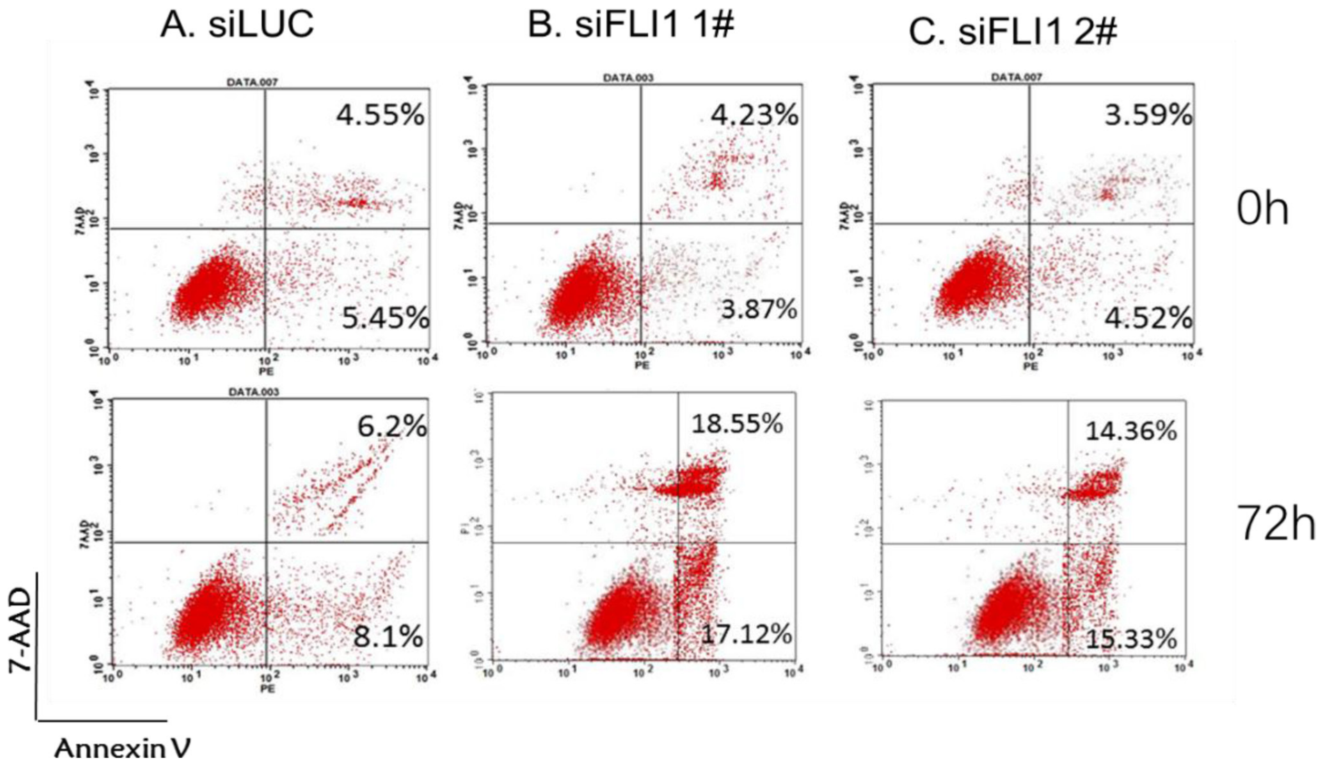

Supplementary Figure 4: ***FLI1*** **knockdown promotes apoptosis in NCI-H1688 cells After knockdown of** ***FLI1*****, NCI-H1688 cells were subject to flow cytometry analysis for cell apoptosis.**

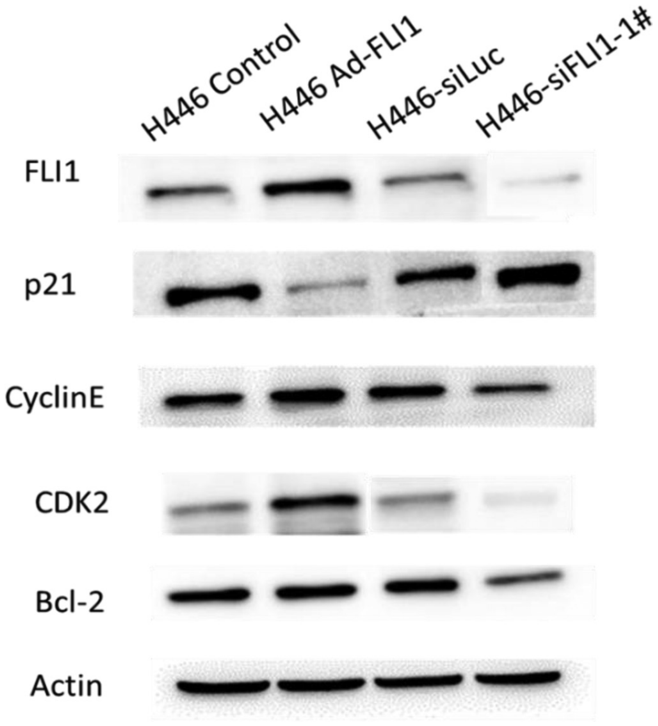

Supplementary Figure 5: Expression of the *FLI1*-miR-17-92 pathway target genes After *FLI1* knockdown, the downstream target genes of the miR-17-92 pathway was quantitated by Western blot. The expression of CyclinE and CDK2 was upregulated when *FLI1* was overexpressed, but was significantly decreased when *FLI1* was knocked down.

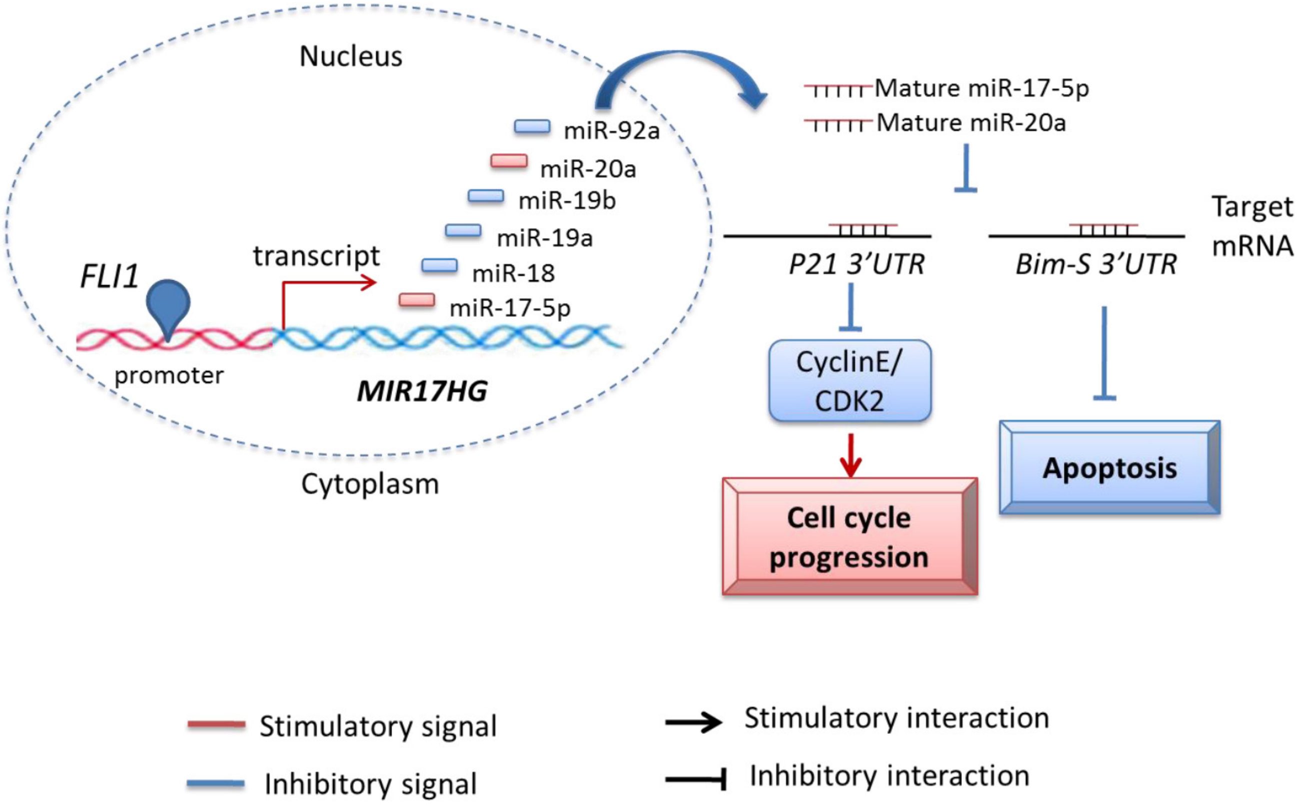

Supplementary Figure 6: The putative model of the *FLI1*-miR17-92 pathway in SCLC. The overexpressed *FLI1* oncoprotein binds to a conserved ETS binding site in the miR-17-92 promoter and activates the transcription of the microRNA cluster in SCLC cells. Two major members of the cluster, miR-17–5p and miR-20a, regulate a large subset of key genes that promote proliferation and cell cycle progression in SCLC. By targeting the downstream *Bim-S* and P21, the oncogenic *FLI1* regulates apoptosis, cell cycle, and finally the development of SCLC.
